# Tumor‐associated macrophages (TAMs)‐derived osteopontin (OPN) upregulates PD‐L1 expression and predicts poor prognosis in non‐small cell lung cancer (NSCLC)

**DOI:** 10.1111/1759-7714.14108

**Published:** 2021-08-22

**Authors:** Yue Li, Hailin Liu, Yujie Zhao, Dongsheng Yue, Chen Chen, Chenguang Li, Zhenfa Zhang, Changli Wang

**Affiliations:** ^1^ Department of Lung Cancer, Tianjin Medical University Cancer Institute and Hospital, National Clinical Research Center for Cancer, Key Laboratory of Cancer Prevention and Therapy Tianjin's Clinical Research Center for Cancer, Tianjin Lung Cancer Center Tianjin China; ^2^ YuceBio Technology Co., Ltd. Shenzhen China

**Keywords:** non‐small cell lung cancer (NSCLC), osteopontin (OPN), PD‐L1, tumor microenvironment, tumor‐associated macrophages (TAMs)

## Abstract

**Background:**

Programmed cell death ligand 1 (PD‐L1) is widely known as an immune checkpoint molecule in tumor cells. Osteopontin (OPN) is expressed by both tumor cells and tumor‐associated macrophages (TAMs), and both autocrine and paracrine of OPN are considered to be involved in tumor metastasis, proliferation and immunosuppression. However, little is known about the relationship between OPN expressed in TAMs (TOPN) and PD‐L1 in non‐small cell lung cancer (NSCLC).

**Methods:**

Tissue microarray was used to detect the expression of TOPN, TAMs and PD‐L1 by multiple quantitative fluorescence staining in 509 NSCLC patients undergoing complete pulmonary resection. The correlations between TOPN, PD‐L1 and clinicopathological data were analyzed. An in vitro coculture system was established to investigate the crosstalk between TOPN and neoplastic PD‐L1. In vivo, the intrinsic features of PD‐L1 in NSCLC xenografts were evaluated after being coinjected with OPN‐positive TAMs, and a series of key cytokines and chemokines were detected in the tumor microenvironment.

**Results:**

A positive association between the TOPN and PD‐L1 expression in tumor tissues from 509 patients with NSCLC was verified. In addition, TOPN and PD‐L1 were independent prognostic factors for overall survival (OS) and disease‐free survival (DFS) of NSCLC patients. Moreover, TOPN upregulated PD‐L1 expression in NSCLC cells through the nuclear factor‐κB (NF‐κB) pathway in vitro TOPN induced the PD‐L1 expression promoted the tumor growth in tumor‐bearing mice, altering immune‐related cytokines and chemokines.

**Conclusions:**

TOPN regulates PD‐L1 expression through the NF‐κB pathway in NSCLS, which is a potential independent biomarker and target for prognosis as well as immunotherapy.

## Graphical abstract

TOPN regulates PD‐L1 expression through NF‐κB pathway in NSCLS, which is a potential independent biomarker and target for prognosis as well as immunotherapy.

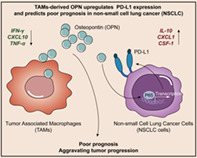

## INTRODUCTION

Although impressive progress including chemotherapy, radiotherapy, and molecular targeted treatment have been made in the management of non‐small cell lung cancer (NSCLC) treatment, the mortality rate of NSCLC is still high, with an overall 5‐year survival rate of only 15%.^1^ Programmed cell death ligand 1 (PD‐L1) as well as programmed cell death‐1 (PD‐1) receptors are two star molecules which are responsible for unleashing T cell‐mediated antitumor immune response. In the past decade, the increasing advances in PD‐1/PD‐L1‐based immunotherapy have provided novel strategies and more selectivity for multiple types of cancer, including NSCLC.^2^, ^3^ The US Food and Drug Administration (FDA) has approved anti‐PD‐1 antibody nivolumab and pembrolizumab for the treatment of advanced‐NSCLC.^4^ Nevertheless, a great improvement in survival rate has only been observed in a small number of NSCLC patients,^5^ as a large number of patients have not obtained benefits, which may be attributed to tumor heterogeneity as well as complexity of the tumor microenvironment.^2^, ^6^ Thus, a better understanding of the relationship between the tumor microenvironment and immune checkpoint molecules is urgently needed to identify novel therapeutic strategies.

Osteopontin (OPN), also known as secreted phosphoprotein 1 (SPP1), has diverse biological functions which are specific to a spectrum of physiological and disease conditions.^7^, ^8^ The main findings of OPN on pathological process are that in inflammation as well as cancer progression, it serves as an indicator of poor prognosis by apoptosis tolerance, which is highly associated with the alteration of host immunity.^9^, ^10^ For example, OPN serves as an immune checkpoint blockade in colorectal cancer, blockade OPN could enhance cytotoxic T lymphocyte lytic activities.^11^ On the other hand, OPN could increase VEGF levels and further facilitate NSCLC progression, which makes it an independent biomarker for predicting NSCLC prognosis.^12^ Hence, it is crucial to gain more insights into the mechanisms of OPN on the tumor microenvironment, including interaction with recognized checkpoint molecules, such as PD‐1/PD‐L1.

In the immune microenvironment, tumor associated macrophages (TAMs) play an important role in immunosuppression.^13^, ^14^, ^15^ It has previously been proven that OPN is expressed by not only tumor cells but in the tumor extracellular matrix, including macrophages (known as TOPN), NSCLC progression intervention, metastasis, as well as immunosuppression.^16^, ^17^, ^18^, ^19^ Nevertheless, the relationship between TOPN and PD‐L1 expression in the NSCLC microenvironment remains unclear. In the present study, we summarize the clinical data from NSCLC patients, which indicate that TOPN level is highly related to the expression of PD‐L1 in NSCLC cells, making TOPN an potential independent biomarker for NSCLC prognosis. In addition, our findings also illustrate that the NF‐κB pathway is essential for TOPN‐mediated PD‐L1 upregulation, which may also consolidate PD‐L1‐related tumor intrinsic features in xenograft models. Our findings provide more insight into the relationship of TOPN and PD‐L1 in NSCLC, providing clues for applying TOPN as a potential therapeutic target and biomarker for immunotherapy in NSCLC.

## METHODS

### Patients

All NSCLC patients undergoing complete pulmonary resection and systematic lymph node dissection at the Cancer Institute and Hospital of Tianjin Medical University (Tianjin, China) from 2004 to 2012 were considered eligible for retrospective analysis of clinical prognostic factors. Patients who were included in the study met the following criteria: (a) a distinctive NSCLC diagnosis based on World Health Organization criteria; (b) The patients had undergone CT or MRI scan for brain, chest and abdomen, and emission computed tomography (ECT) for bone preoperatively to ensure that there was no metastasis; (c) no prior anticancer treatment; (d) the stage II and IIIa patients received adjuvant chemotherapy or radiotherapy, alone or in combination after surgery; (e) complete pulmonary resection and systematic lymph node dissection; (f) complete clinicopathological and follow‐up data. Ethical approval for human subjects was obtained from the research ethics committee of Tianjin Cancer Institute and Hospital, and informed consent was obtained from each patient. Patients who died within 1 month after surgery were excluded from the study. Tumor staging was based on the most recent IASLC TNM classification system.^20^ All patients were followed up until August 31, 2019. Patients who were still alive after the last follow‐up were censored in the study.

### Tissue microarray

Tissues were used to construct a tissue microarray (TMA), as previously described.^16^ To validate the concordance between TMAs and whole tumor sections, we further detected OPN, PD‐L1 and CD68 expression for 50 cases randomly chosen from the 509 patients in comparison with whole tumor sections.

### Multiple quantitative fluorescence staining

Multiple quantitative fluorescence staining of OPN, PD‐L1 and TAMs was performed with monoclonal anti‐rabbit OPN antibody at 1:200 dilution (Abcam, clone ab8448), PD‐L1 antibody at 1:100 dilution (CST, clone E1L3N) and CD68 antibody at 1:100 dilution (Santa Cruz, clone ED1). Multiple quantitative fluorescence staining was performed with the Opal 7‐Color Manual IHC Kit (NEL811001KT, PerkinElmer Inc.) according to the manufacturer's protocol. Nuclei were stained with 4′‐6′‐diamidino‐2‐phenylindole (DAPI, Thermo Scientific) after all the human antigens had been labeled. Negative control slides with the primary antibodies omitted were included in all assays. The stained slides were scanned by the Vectra System (PerkinElmer) to obtain multispectral images.

### Cell cultures

HEK 293 T cells, human NSCLC cell lines (H520 and A549), and mouse macrophage cells (RAW264.7) were purchased from the Institute of Biochemistry and Cell Biology, Chinese Academy of Science (Shanghai, China). All cell lines were cultured in DMEM or RPMI‐1640 medium supplemented with 10% fetal bovine serum (Gibco). The cell lines were used for experiments within 10 passages after thawing.

### Real‐time PCR

Total RNA was extracted from cultured cells, followed with reverse transcription and real‐time PCR as previously described.^21^ For quantification of mRNA levels, GAPDH level was used as an internal control.

### Western blotting

After separation by SDS‐PAGE, the protein was transferred to a PVDF membrane, and blocked with milk in TBST and then incubated with primary antibodies against rabbit anti‐PD‐L1 (CST, clone E1L3N), p65 (Abcam, clone ab32536) or a rabbit anti‐phospho‐NF‐κB p65 (Ser536) (Abcam, clone ab86299). HRP‐conjugated anti‐rabbit antibody (Santa Cruz Biotechnology) was used as secondary antibodies.

### Cytokine and inhibition of the NF‐κB treatments

NSCLC cells were incubated with recombinant human OPN (100 ng/ml, 200 ng/ml, and 400 ng/ml) (R&D Systems) at 37°C for 120 min. NSCLC cells were treated with Helenalin (Abcam, ab146197, 10 ng/ml) for 1 h.

### Immunofluorescence

For immunocytochemistry, cells grown on coverslips were fixed with 4% paraformaldehyde for 15 min and punched by 0.1% Triton X‐100 for 10 min. Cells were then incubated with 3% bovine serum albumin in PBS for 30 min to block the nonspecific binding sites. Cells were incubated overnight with rabbit polyclonal anti‐p65 primary antibody (Abcam, clone ab32536, 1:50). After washing, cells were stained with a matching Alexa Fluor 488‐conjugated secondary antibody (Life Technologies) at RT for 1 h, followed by DAPI staining (Invitrogen). Signals were visualized using a confocal laser scanning microscope.

### Animal studies

Cell line‐derived xenograft (CDX) models were used. Ten female nude mice were randomized into two groups (RAW‐264.7‐OPN/A549, RAW‐264.7‐Ctl/A549) (5 in each group, female BALB/c, 4–6 weeks). A coculture model of macrophages and lung cancer cells in vivo was constructed. Suspensions of 5 × 10^6^ cells A549 and 1 × 10^6^ cells RAW264.7 were injected subcutaneously into the flanks of mice obtained from the Model Animal Research Center of Nanjing University (Nanjing, China). Tumor volume was measured weekly using vernier caliper. The mice were sacrificed, and the tumor tissue was obtained at 5 weeks post‐implantation. Consecutive sections were made for every tissue block of the tumor tissue and stained with hematoxylin–eosin.

### Statistical analysis

The association between variables was analyzed by *χ2* testing analysis. Quantitation of TOPN and PD‐L1 analyses was performed using Bonferroni correction. Overall survival was defined as the interval between the date of surgery and date of death or last follow‐up. Disease‐free survival was defined as the duration between the date of surgery and the date of first recurrence or last follow‐up. OS and DFS were analyzed using the Kaplan–Meier method and compared using the log‐rank test, and multivariate analysis were tested by Cox proportional hazard model. SPSS (version 22.0; IBM Corp) statistical programs were used for all analyses.

## RESULTS

### Clinical characteristics

A retrospective series of 509 NSCLC patients (296 males and 213 females, median age 60 years, range 36–78) was retrieved from the original files of the Tianjin Cancer Institute and Hospital for the study. Follow‐up data were obtained from hospital charts and correspondence with the referring physicians. In this population, the mean follow‐up was 47 months (ranging from 2–187 months), median OS (95% confidence interval [CI]) was 36 (30.8–41.2) months.

### Relationship among TPON and PD‐L1 expression of NSCLC cells and clinicopathological data

**FIGURE 1:**
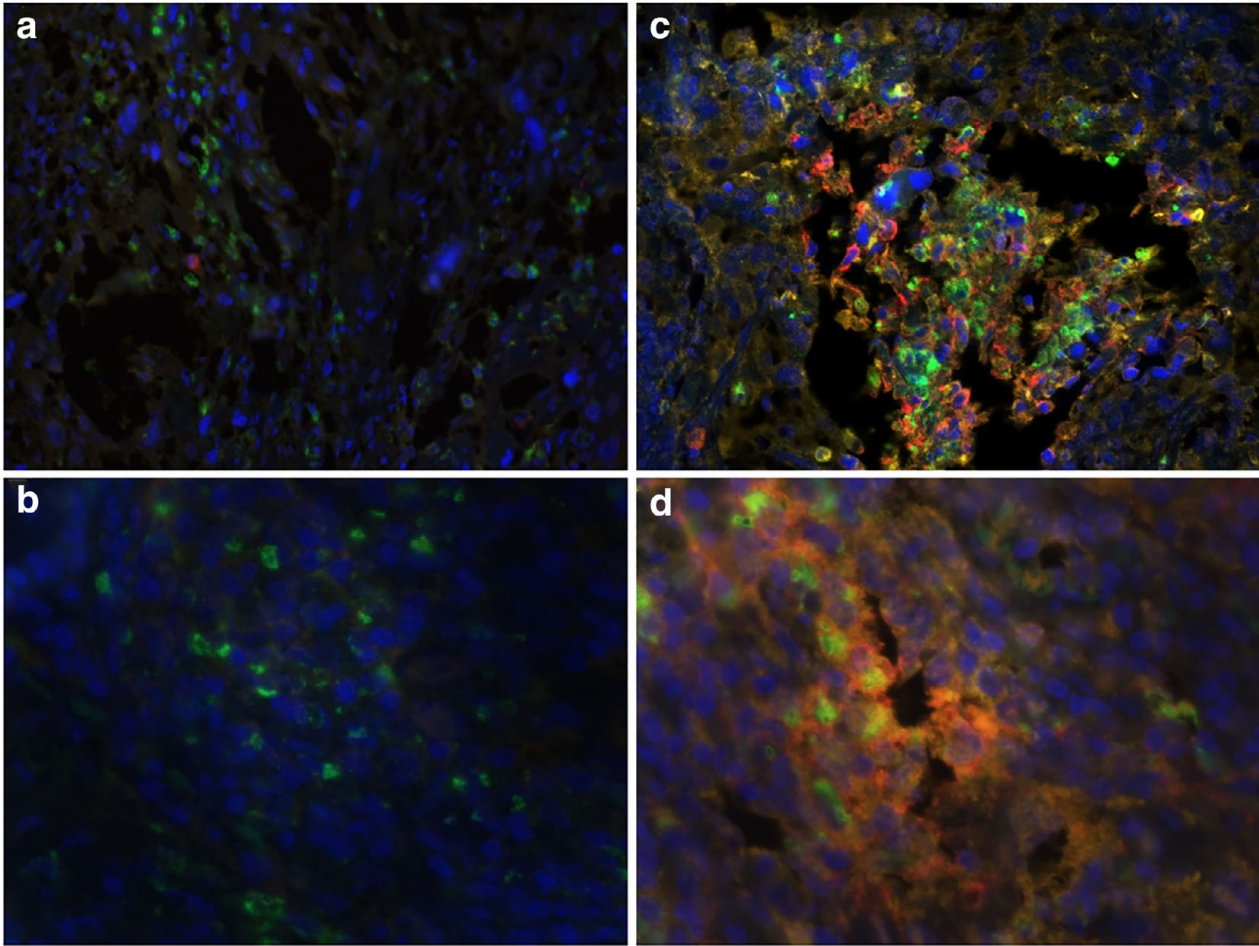
Multiple quantitative fluorescence staining. DAPI (blue), OPN (red), CD68 (green) and PD‐L1 (yellow). OPN expressed by TAMs showed a significant positive correlation with the PD‐L1 expression on NSCLC cells. (a) TOPN‐ with PD‐L1^low^ 20×;(b) TOPN‐ with PD‐L1^low^ 40×; (c) TOPN+ with PD‐L1^high^ 200×;(d) TOPN+ with PD‐L1^high^ 400 ×

The multiplex staining confirmed that OPN‐positive macrophages occurred in 49.1% (250 of the 509 NSCLC patients) (Figure 1(a)). Moreover, among 509 NSCLC patients, 240 patients were observed to have high PD‐L1 expression (Figure 1). To validate the concordance between TMA and whole tumor sections, we further detected OPN, PD‐L1, and TAMs for 50 cases randomly chosen from the 509 patients in comparison with whole tumor sections. We found that TAMs, PD‐L1, and TOPN in the whole tumor sections were 100% (50 of 50) in accordance with the results in the TMAs. We investigated the association of PD‐L1 with clinicopathological features of TOPN expression to elucidate the biological significance. As shown in Table 1, PD‐L1 was associated with recurrence, TNM stage, T stage, N stage, subcarinal lymph node status, number of involved nodes and number of involved nodal stations (*p* < 0.05). Moreover, the multiplex staining results showed that the expression of neoplastic PD‐L1 in tumors with positive TOPN was significantly higher than that in patients with negative TOPN, which revealed a positive association between TOPN and neoplastic PD‐L1 expression (r = 0.4497, *p* < 0.0001) (Figure 1).

**TABLE 1:** Relationship between PD‐L1 and clinicopathological factors of patients

	PD‐L1		
Factors	Low, n (%)	High, n (%)	*p*‐value
Gender			0.0156
Male	143 (28.09%)	153 (30.06%)	
Female	126 (24.75%)	87 (17.09%)	
Age			0.8760
<60 years	133 (26.13%)	117 (22.99%)	
≥60 years	136 (26.72%)	123 (24.17%)	
Recurrence			<0.0001
No	114 (22.40%)	54 (10.61%)	
Yes	155 (30.45%)	186 (36.54%)	
Smoking status			0.0023
Never smoker	129 (25.34%)	83 (16.31%)	
Smoker	140 (27.50%)	157 (30.84%)	
Type of resection			0.9502
Lobectomy	219 (43.03%)	198 (38.90%)	
Pneumonectomy	32 (6.29%)	27 (5.30%)	
Bronchial sleeve resection	18 (3.54%)	15 (2.95%)	
Lesion			0.7667
Central	62 (12.18%)	58 (11.39%)	
Peripheral	207 (40.67%)	182 (35.76%)	
Location of tumor			0.5711
Left	111 (21.81%)	105 (20.63%)	
Right	158 (31.04%)	135 (26.52%)	
Histological subtype			0.0097
Squamous cell carcinoma	68 (13.36%)	86 (16.90%)	
Adenocarcinoma	201 (39.49%)	154 (30.26%)	
Stage			0.0016
I	94 (18.47%)	50 (9.82%)	
II	54 (10.61%)	53 (10.41%)	
IIIA	121 (23.77%)	137 (26.92%)	
T stage			0.0022
T1	123 (24.17%)	81 (15.91%)	
T2	99 (19.45%)	85 (16.70%)	
T3	29 (5.70%)	50 (9.82%)	
T4	18 (3.54%)	24 (4.72%)	
N stage			0.0094
N0	129 (25.34%)	83 (16.31%)	
N1	31 (6.09%)	34 (6.68%)	
N2	109 (21.41%)	123 (24.17%)	
Subcarinal lymph node			0.0009
Negative	222 (43.61%)	168 (33.01%)	
Positive	47 (9.23%)	72 (14.15%)	
Number of involved nodes			0.0005
≤4	208 (40.86%)	152 (29.86%)	
>4	61 (11.98%)	88 (17.29%)	
Number of involved nodal stations			0.0024
<2	179 (35.17%)	128 (25.15%)	
≥2	90 (17.68%)	112 (22.00%)	
TOPN			<0.0001
Negative	194 (38.11%)	65 (12.77%)	
Positive	75 (14.73%)	175 (34.38%)	
TAMs			0.2067
Negative	119 (23.38%)	101 (19.84%)	
Positive	140 (27.50%)	149 (29.27%)	

Abbreviations: TAMs, tumor‐associated macrophages; TOPN, OPN expressed by TAMs; TNM, tumor–node–metastasis.

### Neoplastic TOPN was significantly associated with poor prognosis in NSCLC patients

**FIGURE 2:**
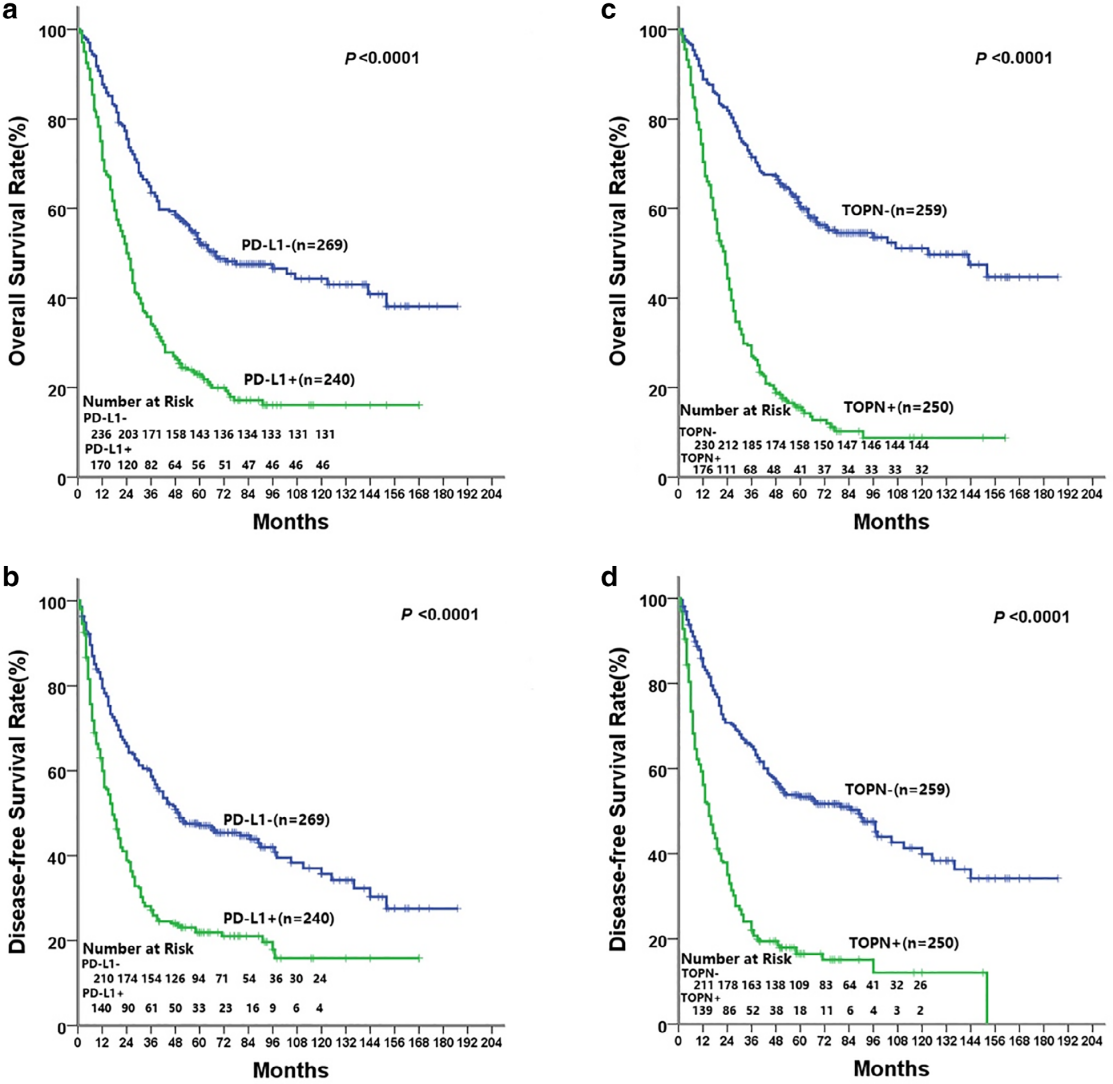
Prognostic significance was assessed using Kaplan–Meier survival estimates and log‐rank tests. Comparisons of OS and DFS by PD‐L1 (a, b) and TOPN (c, d)

The 5‐, 8‐, and 10‐year overall survival rate (OS) was 39.10%, 35.17% and 34.77%, the 5‐, 8‐, and 10‐year disease free survival rate (DFS) was 24.95%, 8.84% and 5.50%, for the total study population, respectively. Analyzed by the Kaplan–Meier log‐rank test, both TOPN expression and PD‐L1 were significant prognostic factors for NSCLC. Among 5‐, 8‐, and 10‐year OS as well as median survival time (MST) of NSCLC patients with higher PD‐L1 expression were significantly lower than those in the group with lower PD‐L1 expression (23.33% vs. 53.16%, 19.17% vs. 49.44%, 19.17% vs. 48.70%, 24 months vs. 55 months, *p* < 0.0001, Figure 2(a)). The 5‐, 8‐ and 10‐year DFS were 13.75%, 3.75% and 1.67% for PD‐L1‐higer group, 34.94% 13.75% and 8.92% for PD‐L1‐lower group, and MST were 17 and 45 months for each subgroup (*p* < 0.0001; Figure 2(b)). Meanwhile, both 5‐, 8‐ and 10‐year OS and MST in TOPN positive group were significantly lower than those in the TOPN negative group (16.40% vs. 61.00%, 13.20% vs. 56.37%, 12.80% vs. 55.60%, 24 months vs. 60 months, *p* < 0.0001; Figure 2(c)). The 5‐, 8‐ and 10‐year DFS was 7.20%, 1.60% and 0.80%, respectively for the TOPN positive group, while 42.08%, 15.83%, and 10.04%, respectively for the TOPN negative group (*p* < 0.0001; Figure 2(d)). Multivariate analysis indicated that overexpression of PD‐L1 was an independent risk factor for both OS and DFS. These findings suggest PD‐L1 and TOPN have the potential to serve as prognostic indicators for patients with NSCLC (Table 2).

**TABLE 2:** Prognostic factors for DFS retained multivariate analysis in 509 NSCLC patients

	OS		DFS		
Variables	HR (95.0% CI)	*p*‐value	*HR*	95.0% CI	*p*‐value
TNM	1.114 (1.012–1.226)	0.027	1.368 (1.021–1.832)		0.036
T stage	0.765 (0.519–1.128)	0.176	1.072 (0.972–1.183)		0.166
N stage	1.623 (1.119–2.254)	0.011	1.494(1.037–2.154)		0.031
Subcarinal lymph node	1.264 (0.947–1.689)	0.112	0.817 (0.0.559–1.195)		0.297
Number of involved nodal station	1.445 (0.976–2.140)	0.066	1.415 (0.946–2.118)		0.091
Number of involved nodes	0.918 (0.656–1.285)	0.619	0.972 (0.689–1.371)		0.873
TOPN	2.732 (2.110–3.537)	<0.0001	2.176 (1.685–2.811)		<0.0001
PD‐L1	1.495 (1.172–1.908)	<0.001	1.330 (1.038–1.703)		0.024

Abbreviations: CI, confidence interval; DFS, disease free survival; HR, hazard ration; OS, overall survival; TNM, tumor–node–metastasis; TOPN, OPN expressed by TAMs.

### PD‐L1 expression in NSCLC cells is enhanced after coculture with OPN‐expressing macrophages

**FIGURE 3:**
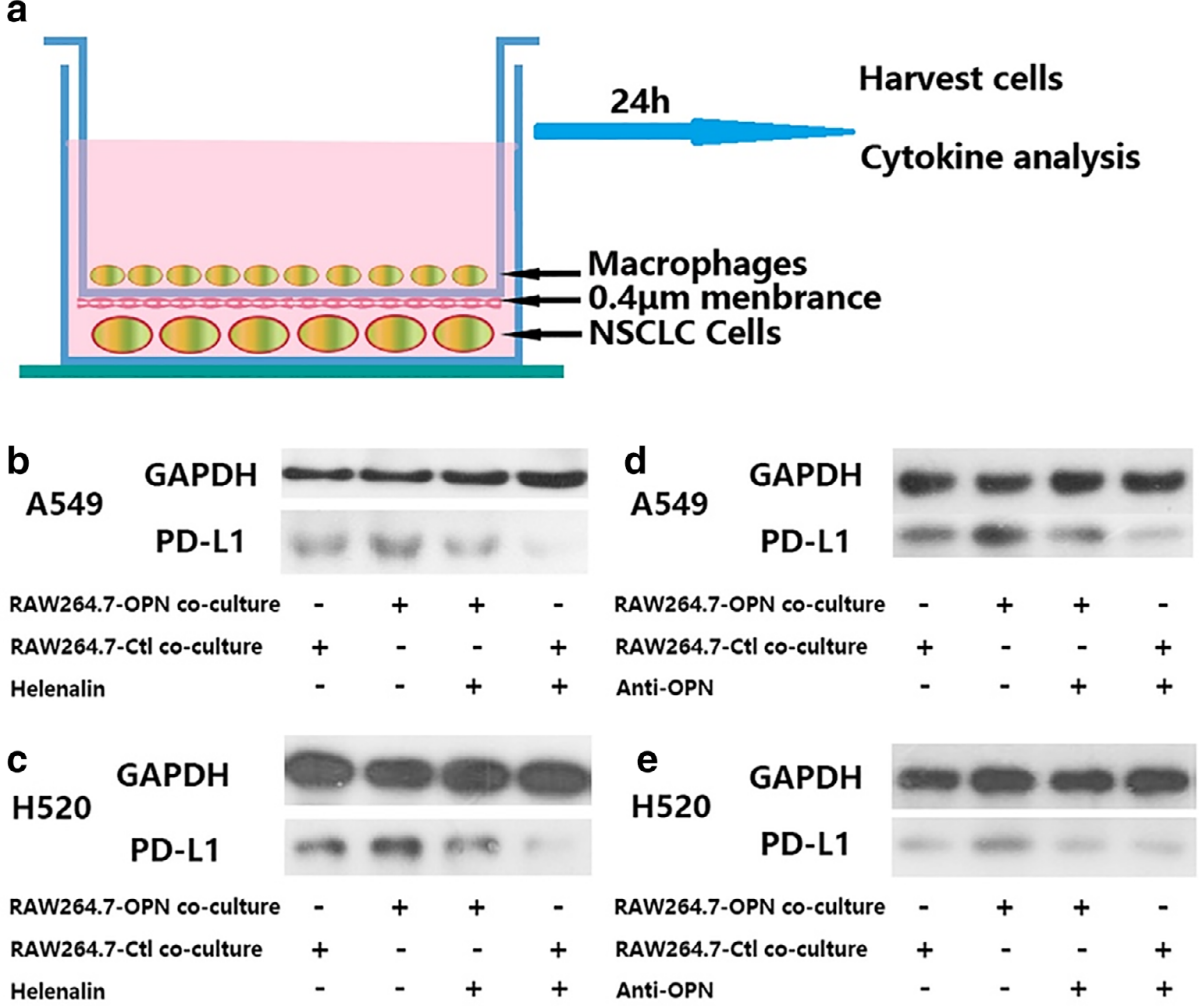
(a) Schematic showing the NSCLC cells cocultured with macrophages of different OPN levels in a transwell apparatus of 0.4 μm pore size; (b, c, d and e) A549 and H520 cells were treated with helenalin (Abcam, ab146197, 10 ng/ml) for 1 h or monoclonal anti‐rabbit OPN antibody, and then were cocultured with macrophages (RAW264.7‐Ctl or RAW264.7‐OPN) for 24 h. Western blot analysis of PD‐L1 expression in different cococulture groups

To further validate the positive correlation between TOPN and neoplastic PD‐L1, we first established an in vitro coculture system by culturing NSCLC cells with different OPN expression levels RAW264.7 macrophage cells (Figure 3(a)). We then assessed the PD‐L1 expressed by NSCLC cells. After coculture with macrophages, neoplastic PD‐L1 was expressed, which was cocultured with RAW264.7‐OPN, and was significantly higher than the RAW264.7‐Ctl group (Figure 3(b)–(e)). These results strongly suggested that OPN may influence neoplastic PD‐L1 expression via macrophages.

### OPN induces of PD‐L1 expression in NSCLC cells via regulating the NF‐κB/p65 pathway

**FIGURE 4:**
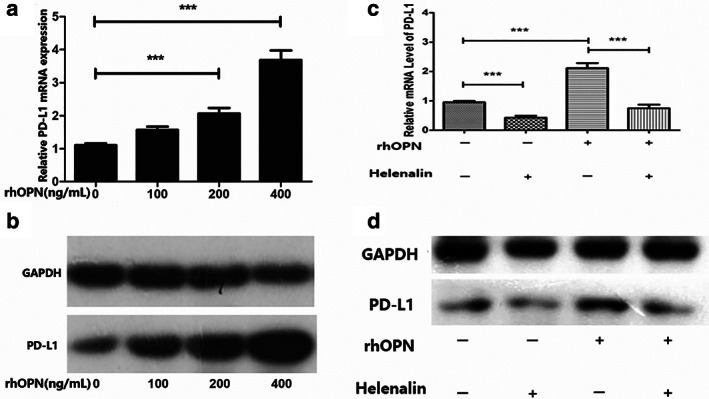
NSCLC cells (A549) were incubated with of recombinant human OPN (100 ng/ml, 200 ng/ml, 400 ng/ml) (R&D systems) at 37°C for 120 min. mRNA and protein were examined by RT‐PCR (a) and Western blot (b); NSCLC cells were treated with helenalin for 1 h and then incubated with of recombinant human OPN (200 ng/ml), RT‐PCR(c) and Western blot (d) were examined

To gain more insight into the mechanism of action on the pathways by which OPN regulates PD‐L1 expression, the NSCLC cell lines were treated with recombinant human OPN (rhOPN). The PD‐L1 expression in both mRNA and protein levels significantly increased after treatment with recombinant human OPN, in a dose‐dependent manner (Figure 4(a) and (b), *p* < 0.001). It is hypothesized that OPN regulates PD‐L1 expression through nuclear transcription pathways for which OPN could activate the NF‐κB/p65 pathway^22^and NF‐κB directly or indirectly.^23^ This hypothesis was verified by blocking the NF‐κB/p65 pathway using helenalin. Western blot and RT quantitative PCR showed that helenalin could reverse the upregulation of PD‐L1 induced by OPN (Figure 4(c) and (d), *p* < 0.001).

**FIGURE 5:**
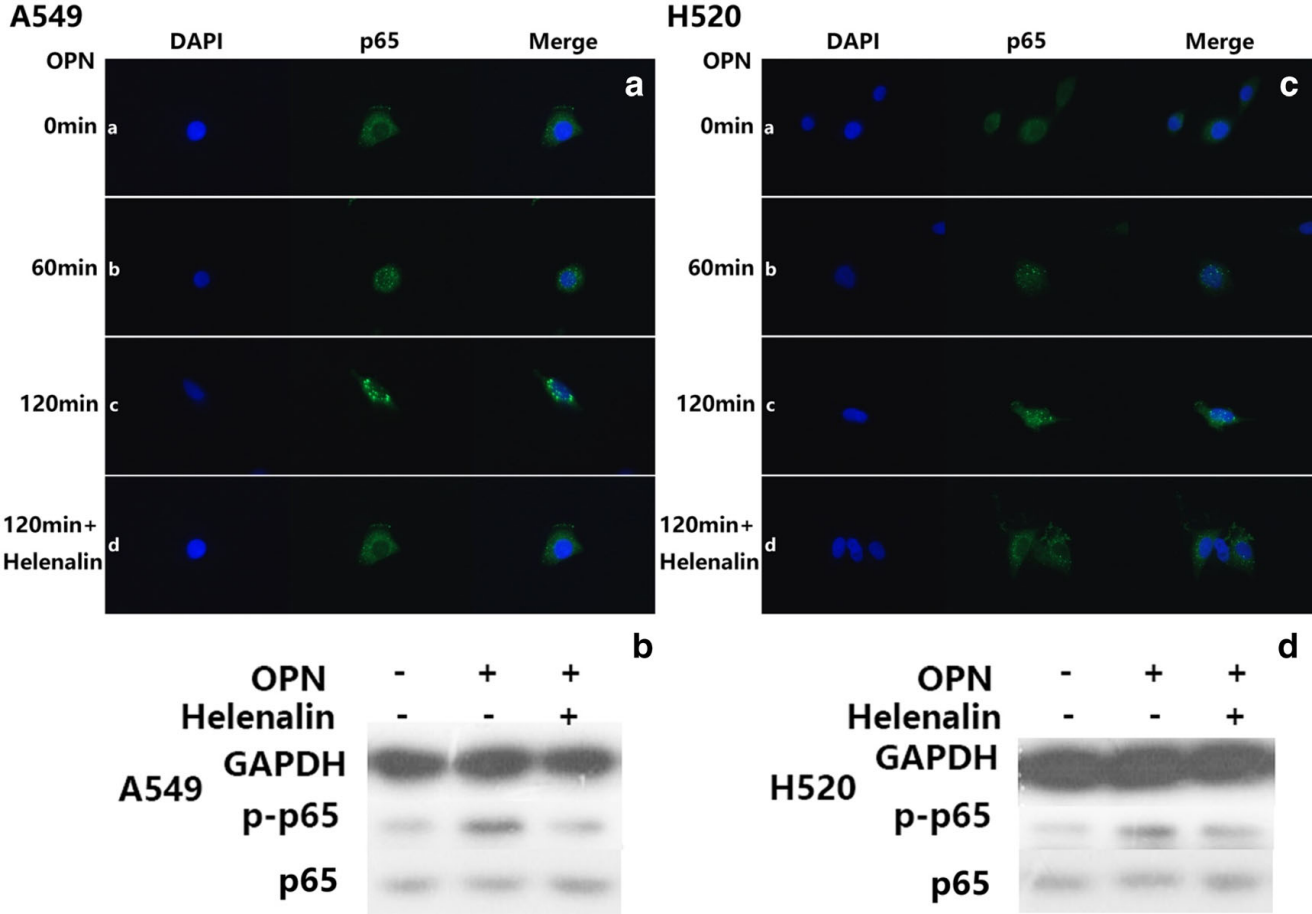
Immunofluorescence assay for the localization of p65 protein in A549 (a) and H520 cells (c). A549 and H520 cells grown on glass slides were treated with 200 ng/ml of OPN (for 0–120 min). p65 was mainly located in the cytoplasm of A549 and H520 cells before OPN treatment (a). From 60 min (b) and 120 min (c) after OPN treatment, translocation of p65 into the nucleus was observed and increased; p65 was completely accumulated in the nucleus of NSCLC cells after OPN treatment for 120 min (c). The addition of helenalin can reverse the upregulation effect of OPN on translocation of p65 into the nucleus (d). Western blot analysis of proteins detected by probing with anti‐p65 or anti‐phosphorylated p65 antibodies. Recombinant human OPN increased the level of phosphorylated p65 (b, d)

To further examine the effect of OPN on the p65 subunit of NF‐κB, both A549 and H520 cells were treated with 200 ng/ml recombinant human OPN and helenalin in medium at 37°C for 0 to 120 min. After treatment with recombinant human OPN, increased translocation of p65 from cytoplasm to nucleus was observed (Figure 5(a) and (c)). We also examined the nuclear expression of p‐p65 by Western blot and found that p65 had cytoplasmic transfer to the nucleus, while p65 had no quantitative change (Figure 5(b) and (d)).

After coculture with macrophages, the PD‐L1 expression level in NSCLC cells (both A549 and H520) cocultured with macrophages was significantly higher in the RAW264.7‐OPN group than that in the RAW264.7‐Ctl group (Figure 3). After the application of NF‐κB inhibitor, it was shown that helenalin could reverse the upregulation of neoplastic PD‐L1 induced by TOPN (Figure 3(b) and (c)). Similarly, the addition of OPN antibody could reverse the upregulation effect of TOPN on PD‐L1 (Figure 3(d) and (e)).

### OPN‐expressing macrophages upregulate PD‐L1 expression of NSCLC cells and aggravate tumor progression

**FIGURE 6:**
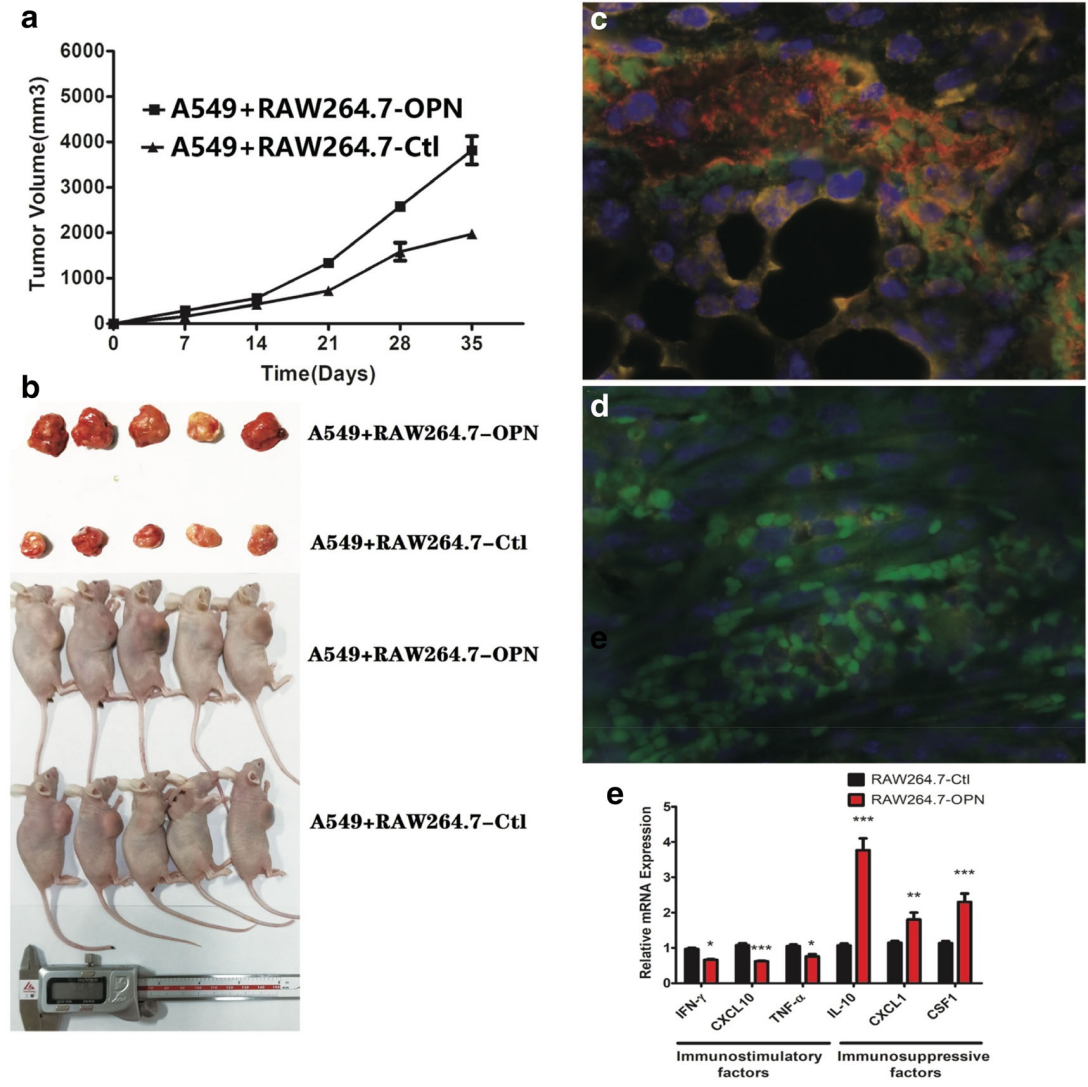
The effect of TOPN on in vivo tumor growth and the PD‐L1 expression of A549 cells. (a, b) In vivo tumor growth in RAW‐264.7‐OPN mice was significantly greater compared with the RAW‐264.7‐Ctl group in tumor volume. The NSCLC cells A549, which were cocultured with RAW‐264.7‐OPN, exhibited an increase in the level of PD‐L1 (c) compared with the A549/RAW‐264.7‐Ctl cocultured group (d). Relative expression of the indicated genes by qPCR(E). Data shows the mean ± SEM (*n* = 6)

Subsequently, we evaluated the intrinsic features of TOPN‐influenced PD‐L1 in vivo. The results showed that in the RAW‐264.7‐OPN/A549 coculture group, much larger histological lesions were observed compared with that of the RAW‐264.7‐Ctl/A549 coculture group (Figure 6(a),and (b)) (3817 ± 311.4 mm^3^ vs. 1497 ± 54.98 mm^3^, *p* < 0.001). Moreover, the multiplex staining results showed that after being coplanted with RAW‐264.7‐OPN in vivo, the PD‐L1 level in NSCLC cells A549 increased significantly (Figure 6(c)) than the A549/RAW‐264.7‐Ctl cocultured group (Figure 6(d)). In addition, we further evaluated the expression of representative cytokines and chemokines, including the immunosuppression and immunostimulatory effects. The interferon‐γ (IFN‐γ), tumor necrosis factor α (TNF‐α), and C‐X‐C motif chemokine ligand 10 (CXCL10) were markedly reduced in the RAW‐264.7‐OPN/A549 group. Furthermore, a significant reduction was found in IL‐10 expression, CSF1, as well as CXCL1, which were associated with M2 macrophage polarization (Figure 6(e)). These results further verify that TOPN could alter the intrinsic features of NSCLC by regulating PD‐L1 expression.

## DISCUSSION

In the present study, our main finding was that OPN produced by TAMs upregulates PD‐L1 expression and predicts poor prognosis among NSCLC patients. Further, our in vitro and in vivo results indicate that TOPN regulates PD‐L1 through the NF‐κB pathways, which ultimately alters PD‐L1‐related tumor intrinsic features. As a phosphorylated glycoprotein, OPN is involved in a series of biological functions, such as promoting cell adhesion, cell migratory activity, bone metabolism, and stone formation. Moreover, in pathological processes, OPN also serves as a prominent tumor‐sustaining inflammatory mediator, which is expressed not only by various malignant tumors but also by macrophages, related to tumor metastasis, proliferation and immunosuppression.^24^ In glioblastoma, for example, OPN ws found to maintain the gene characteristics and phenotype of M2 macrophages, which contribute significantly to glioblastoma progression. In addition, OPN deficiency initiated immune reaction, and resulted in a marked decrease in M2 macrophages and a significant increase in T cell effector activity in gliomas.^25^ In addition, the relationship between OPN and PD‐L1 has been also documented. In lung adenocarcinoma, upregulation by OPN induces macrophage polarization which facilitates immune escape.^26^ In hepatocellular carcinoma (HCC), the OPN/CSF1/CSF1R axis plays a critical role in the immunosuppressive nature of the HCC microenvironment. However, few studies have illustrated the potential role of macrophage‐derived OPN on PD‐L1 in NSCLC.

The infiltrating immune cells, such as TAMs,^27^, ^28^ produced immune suppressive factors. OPN could also be expressed by TAMs, known as TOPN. Although we have previously reported that TAM infiltration was not an independent prognostic factor of NSCLC, without correlation of tumor metastasis,^16^ it is unclear whether TOPN directly regulates neoplastic PD‐L1, and serves as an independent biomarker indicating prognosis of NSCLC, which still remains to be uncovered. In the present study, multiple quantitative fluorescence staining was conducted to evaluate a clinical NSCLC data set, which indicated that TOPN was positively correlated with the expression of PD‐L1 in NSCLC patients. This finding is crucial, for it is presumed that partial TAMs have a regulatory effect on PD‐L1, which further indicates that TOPN is an potential independent regulatory factor on PD‐L1 expression.

Previously, Chen et al.^29^ reported that, examined by IHC, positive PD‐L1 expression in primary cancer cells was found in 136 (65.3%) patients, which were negatively correlated with lymph node metastasis, which is similar to the study by Velcheti et al.^30^ In the present study, the OS as well as MST in the TOPN positive group was significantly lower than those in the TOPN negative group, and DFS in the TOPN positive group was significantly longer than that in TOPN negative group, suggesting that TOPN is a prognosis biomarker independent of PD‐L1.

Previous studies have shown that the expression of PD‐L1 in tumor cells can be regulated by many factors and molecular pathways in the microenvironment. As for nonimmune cells, PD‐L1 can be induced by IFN‐γ and TNF‐α on endothelial cells.^31^ Moreover, microenvironment cells, including macrophages can also regulate PD‐L1 expression in multiple types of cancer. For example, in the HCC microenvironment, macrophages induced upregulation of PD‐L1 expression through NF‐κB and STAT3 pathways, suggesting that the overexpression of PD‐L1 in HCC may be mediated by inflammatory microenvironment involving macrophages.^32^ Likewise, PD‐L1 expression on PDAC cells was also positively related with macrophage infiltration in tumor stroma and infiltrating macrophages derived TNF‐α upregulated PD‐L1 expression on PDAC cells via NF‐κB pathway.^21^ In addition, Lim et al.^33^ showed that COP9 signalosome 5 (CSN5), induced by NF‐κB p65, is required for TNF‐α‐mediated PD‐L1 stabilization in breast cancer cells. These studies indicate the significant role and related pathways that macrophages regulate PD‐L1 expression. However, it still remains to be determined whether TOPN could regulate PD‐L1 expression, as well as the potential mechanism of TOPN on PD‐L1 expression. In our subsequent coculture model, we verified that TOPN significantly induced PD‐L1 expression both in vitro and in vivo. As NF‐κB pathways are essential for macrophage action on PD‐L1 expression, it is supposed that TOPN may alter PD‐L1 levels through NF‐κB pathways. We hence conducted further validation by using the NF‐κB inhibitor helenalin, and the results indicated that NF‐κB pathways are crucial for TOPN‐mediated PD‐L1 upregulation.

In the tumor microenvironment, TAMs are the major source of the inflammatory cytokines.^34^, ^35^ Depending upon their microenvironment, TAMs mainly polarize toward “M1”, which secret TNF‐α, IL‐12, and promote antitumor resistance, or “M2” phenotype that secret IL‐10, TGF‐β, and play vital roles in angiogenesis and tumor progression.^36^ In nude mice model, TOPN could also promote the growth of tumor cells and induce the expression of PD‐L1 of NSCLC. In addition, in the RAW264.7‐OPN group, the mRNA level of tumor suppressor immunostimulatory factors decreased, while the level of tumor immunosuppressive factors increased, indicating that TOPN could promote tumor progression in the tumor microenvironment. OPN could be a marker for defining M2 TAMs, and could be a new unfavorable factor to discriminate biological functions of TAMs.

The next frontier of immunotherapy is to find ways to improve the efficacy of checkpoint inhibitors through combined approaches, especially in cancer with limited clinical efficacy. Recent studies have shown that the combination of anti‐PD‐1/PD‐L1 and CSF‐1R inhibitor,^37^ TGF‐β inhibitor^38^ and blocking FcγR on macrophage^39^ can synergistically enhance antitumor immunity. In view of the important role of TOPN in tumor progression, metastasis, immunosuppression and regulation of PD‐L1, the treatment methods for TOPN need to be developed urgently in order to achieve better effect in combination with anti‐PD‐1/PD‐L1. In addition, based on our clinical data analysis, the TOPN level in NSCLC patients could also serve as a potential independent biomarker for evaluating and predicting the efficacy of immunotherapy, including the administration of immune checkpoint inhibitors.

In conclusion, TOPN would be unfavorable prognostic factors for NSCLC patients, who underwent curative resection. TOPN can induce tumor‐mediated secretion of PD‐L1 via the NF‐κB/p65 pathway. Thus, timely treatment for NSCLC patients with TOPN+ phenotype should be undertaken to reduce tumor metastasis and prolong survival. Moreover, TOPN may be a new factor in discriminating the biological functions of TAMs. The combination of blocking OPN on macrophages and anti‐PD‐1/PD‐L1 might shape the future of cancer immunotherapy.

## CONFLICT OF INTEREST

The authors declare that the research was conducted in the absence of any commercial or financial relationships that could be construed as a potential conflict of interest. All the authors declared that there is no conflict of interest.
